# Loneliness in adolescents with different social media behavior

**DOI:** 10.1192/j.eurpsy.2022.1114

**Published:** 2022-09-01

**Authors:** E. Zakirova, N. Poskrebysheva

**Affiliations:** Moscow Lomonosov State University, Faculty Of Psychology, Moscow, Russian Federation

## Abstract

**Introduction:**

Loneliness is a critical issue of adolescents that has grown severe during the last decade. Social media use is often regarded as negative factor of loneliness experience in connection with escapism. At the same time social media is an important part of adolescents’ communication sphere. The present study aims to explore positive functions of social media which can help adolescents cope with loneliness.

**Objectives:**

The present research studies features of loneliness representation in adolescents with different behavior in social media.

**Methods:**

Multidimensional Inventory of Loneliness Experience; Cognitive Processing of Social Information in Internet Questionnaire; Method of unfinished sentences about loneliness and social media; Questionnaire about social media were used in the study with 44 adolescents, aged from 13 to 18.

**Results:**

Adolescents have a higher level of loneliness (M = 29.6) than the results in 2013 (M = 17.7) show. Context analysis of unfinished sentences shows that 21% of adolescents have a various representation of loneliness, 71% perceive loneliness as negative. General feel of loneliness tends to show negative correlations with adequate perception of information in social media (k = - 0.317; p = 0.038). Negative attitude to loneliness is negatively correlated with interpretation adequacy of social media content (k=-0.568, p<0,001). Adolescents with low levels of holistic social media perceptions have higher levels of loneliness. Use of social media can reduce feelings of loneliness (p = 0.002).

**Conclusions:**

Social media expand adolescents’ representation of loneliness. Productive use of social media can help adolescent cope with loneliness.

**Disclosure:**

No significant relationships.

